# Cognitive behavioural therapy (CBT) for anxiety in people with dementia: study protocol for a randomised controlled trial

**DOI:** 10.1186/1745-6215-13-197

**Published:** 2012-10-23

**Authors:** Aimee Spector, Martin Orrell, Miles Lattimer, Juanita Hoe, Michael King, Kate Harwood, Afifa Qazi, Georgina Charlesworth

**Affiliations:** 1 Department of Clinical, Educational and Health Psychology, University College London, 1-19 Torrington Place, London, UK; 2 Department of Mental Health Sciences, University College London, Charles Bell House, 67-73 Riding House Street, London, UK; 3 Research and Development Department, North East London NHS Foundation Trust, Goodmayes Hospital, Barley Lane, London, Ilford, Essex IG3 8XJ, UK; 4 61 St Augustine’s Road, London, NW1 9RR, UK

**Keywords:** Anxiety, Alzheimer’s, Dementia, Carers, CBT, Cognitive behavioural therapy, Psychosocial intervention, Randomised controlled trial (RCT)

## Abstract

**Background:**

Many people with dementia experience anxiety, which can lead to decreased independence, relationship difficulties and increased admittance to care homes. Anxiety is often treated with antipsychotic medication, which has limited efficacy and serious side effects. Cognitive behavioural therapy (CBT) is widely used to treat anxiety in a range of populations, yet no RCTs on CBT for anxiety in dementia exist. This study aims to develop a CBT for anxiety in dementia manual and to determine its feasibility in a pilot RCT.

**Methods/design:**

Phase I involves the development of a CBT for anxiety in dementia manual, through a process of (1) focus groups, (2) comprehensive literature reviews, (3) expert consultation, (4) a consensus conference and (5) field testing. Phase II involves the evaluation of the manual with 50 participants with mild to moderate dementia and anxiety (and their carers) in a pilot, two-armed RCT. Participants will receive either ten sessions of CBT or treatment as usual. Primary outcome measures are anxiety and costs. Secondary outcome measures are participant quality of life, behavioural disturbance, cognition, depression, mood and perceived relationship with the carer, and carer mood and perceived relationship with the person with dementia. Measures will be administered at baseline, 15 weeks and 6 months. Approximately 12 qualitative interviews will be used to gather service-users' perspectives on the intervention.

**Discussion:**

This study aims to determine the feasibility of CBT for people with anxiety and dementia and provide data on the effect size of the intervention in order to conduct a power analysis for a definitive RCT. The manual will be revised according to qualitative and quantitative findings. Its publication will enable its availability throughout the NHS and beyond.

**Trial registration:**

ISRCTN64806852

## Background

In the UK, over 700,000 older people have dementia, placing an enormous strain on public services and family carers [1]. There are limits in the effectiveness and use of dementia medication [2] and in recent years there has been an increase in the development of psychosocial treatments for dementia [3]. Anxiety is common in dementia, with prevalence estimated from 5-21% for anxiety disorders and up to 71% for anxiety symptoms [4]. In addition to excessive worries and fears, anxiety may physically manifest as agitation, motor restlessness, day/night disturbance and aggression. It has been associated with high physical dependency and problems in the patient/carer relationship [5], decreased independence and limitations in activities of daily living [6], increased behavioural problems [7] and increased admissions to nursing care [8]. Anxiety is often treated with antipsychotic medication, which has limited efficacy and serious side effects, including sedation, depression, stroke and cognitive deterioration [9].

Cognitive behaviour therapy (CBT) [10] focuses on the interplay among people’s thoughts, feelings and behaviour. It primarily focuses on the here and now, is highly collaborative and aims to identify personalised, time-limited goals and strategies that are put into practice between sessions (“homework”). There is robust evidence that CBT is an effective first-line strategy for anxiety in general adult populations [2] and for cognitively intact older people [11]. CBT has been widely adopted by the NHS, for example ‘Improving Access to Psychological Therapies’ (IAPT) [12] aims to support primary care trusts in implementing CBT for depression and anxiety in people of 'working age'.

People with dementia have diminished cognitive resources, but there is evidence that they can learn and develop skills, even when moderately cognitively impaired [13]. This suggests that CBT in an adapted form could be used with this client group, as it has been in other impaired populations such as those with learning disabilities [14]. There is evidence for the effectiveness of CBT for depression in dementia, primarily through case studies and an RCT by Teri et al. [7]. There are only four small studies examining CBT for anxiety in dementia. Kipling et al. [15] found changes in behaviour, cognition and mood in a group of three people. Koder [16] and Kraus et al. [17] each described two individual cases and found clinically meaningful reductions in anxiety, plus improved mood and participation in pleasurable activities [17], and reduction in alcohol intake and night awakenings [16]. Paukert et al. [18] developed Peaceful Mind, a CBT intervention for anxiety and dementia, finding reductions in anxiety, depression and carer distress in an open trial containing eight individuals. All four studies suggest that CBT in people with dementia is feasible and concluded that larger trials are now needed, yet there are no published RCTs in CBT for anxiety for dementia.

There will be two phases to this trial, which correspond to phase I and II of the MRC’s guidelines for developing a complex intervention and assessing feasibility [19]. The key aims are:

(1) To develop a CBT intervention manual.

(2) To assess the feasibility of the intervention through a single-blind, pilot RCT on CBT versus treatment as usual (TAU) for people with dementia and their carers. This will include an assessment of acceptability, compliance, recruitment and retention.

(3) To conduct qualitative interviews with participants, assessing the acceptability of the treatment and examining whether changes generalised.

## Method

### Phase I: Development phase

This phase consisted of four stages:

1. *Focus groups:* We conducted nine focus groups with service-users (people with dementia, family carers and staff groups) on causes and management of anxiety [20]. Emerging themes included coming to terms with the diagnosis and loss of skills as causes, and person-centred care, memory aids and medication as a last resort in terms of management.

2. *Comprehensive literature reviews:* We have published the protocol for a Cochrane systematic review on psychosocial interventions for anxiety and depression in dementia [21] and are currently writing the full review. The findings of relevant studies identified in the Cochrane review along with the focus group findings and outcomes of a comprehensive review of the literature on CBT for anxiety (in general populations and with older adults) were used to adapt the best features and identify key techniques and potential adaptations and generate version 1 of the manual.

3. *Expert consultation*: Version 1 of the manual was sent to three experts in dementia and/or CBT and revised according to their comments, generating version 2.

4. *Consensus process:* A Delphi process [22] is commonly used to reach consensus when defining new interventions in health service research. We used a modified Delphi process, outlining a set number of cycles at the outset. Version 2 was brought to an advisory panel of 30 multidisciplinary experts (psychologists, psychiatrists, CBT therapists, carers, admiral nurses and occupational therapists) at a half-day consensus conference. Different parts of the manual were discussed by small groups, with the aim of generating feedback and suggestions for modifications. Amendments following the consensus conference generated version 3 of the manual.

5. *Field testing:* Version 3 was field tested with three people with dementia and their carers, which generated information about modifications needed to improve its feasibility and relevance in practice. These data were used to develop version 4 of the manual, which was sent back to an expert panel for further comments. Further modifications resulted in version 5, to be used for the trial.

### Phase 11: Intervention phase

#### Design

**Figure 1:**
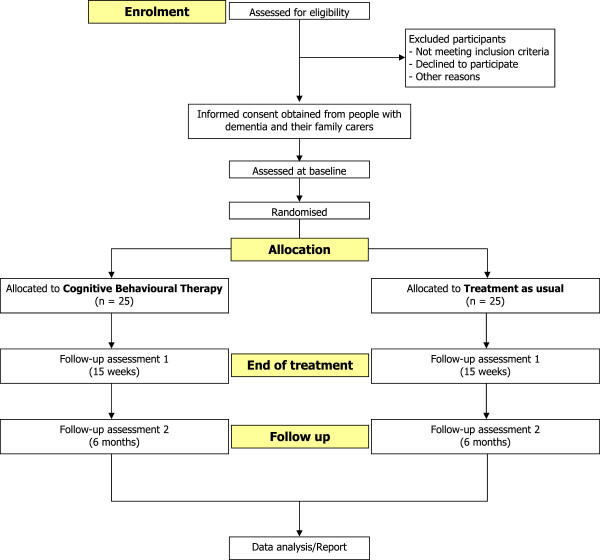
CBT for anxiety in people with dementia flow diagram.

The design is a single-blind, multicentre, pilot RCT of CBT plus treatment as usual (TAU) versus TAU for people with dementia. Patient-carer dyads will be randomly allocated to either CBT or TAU. As no trials have been done in this area, we were unable to estimate the likely effect size of this intervention. We will recruit 50 participants with power set at 80%, *p* = 0.05. This will be adequate to detect an effect size of 0.8 or above. An objective of this study is to provide data on the actual effect size of the intervention in order to do a power analysis for a definitive RCT. Figure 1 shows the trial design.

#### Participants

Fifty participants and their carers will be recruited through North East London Foundation Trust (NELFT) and North Essex Partnership NHS Foundation Trust (NEPFT). Recruitment will be through memory services, community mental health teams, admiral nursing teams and outpatient referrals.

Inclusion criteria are:

1. Meet DSM-IV criteria for dementia in mild to moderate range, Clinical Dementia Rating (CDR) [23] score of 0.5, 1 or 2.

2. Clinical anxiety, as determined by a score of 11 or above on the Rating Anxiety in Dementia scale (RAID) [24].

3. Living in the community.

4. The presence of a carer who is willing to participate in the therapy.

5. An ability to understand and communicate in English.

6. Willing to engage in therapy involving discussion of thoughts and feelings.

Exclusion criteria are:

1. Co-morbid psychiatric disorder (e.g. psychosis) or challenging behaviour (e.g. severe agitation) likely to prevent engagement in therapy.

2. Presence of a learning disability or severe physical illness that could impact on participation.

#### Ethical considerations

Ethical approval was obtained through the East London 3 Research Ethics Committee (ref. no. 10/H0701/124). Informed consent will be sought from participants and their carers, using current guidance from the British Psychological Society on evaluation of capacity. Participants may withdraw from the trial at any time without giving a reason. The withdrawal of the participant from the trial will not affect their access to other appropriate treatments or services.

All information disclosed in the study will be kept confidential, and participants will not be identifiable in any material published as part of the study in any way. All data are stored without any identifying details under secure conditions and all tapes will be destroyed at the end of the trial. In line with Standard Operating Procedures for the study, the reporting of Serious Adverse Events will be made to the Chief Investigator. The Chief Investigator will then inform the Research & Development Manager and Trust Governance Manager. Local policy and procedures will be followed for reporting and investigating serious untoward incidents.

#### Randomisation

Randomisation will be conducted by telephoning an independent administrator within PRIMENT Clinical Trials Unit after the participant/carer has provided informed consent and baseline data. The randomisation schedule is being developed by an independent statistician in PRIMENT, and blocking will be employed (with block sizes varying between 4 and 6) to help ensure equal numbers in the intervention and control arms.

#### Interventions

CBT plus TAU: The patient-carer dyads will participate in 10 weekly sessions, each lasting 1 h hour. This number was determined on the basis of the published literature, the team’s experience and patient and carer feedback.

The manual is divided into four phases. Phase 1 involves assessment and formulation. Key aims are to build a collaborative relationship, socialisation to the CBT model, identifying goals and establishing the involvement of the carer. The carer’s role is to support the person with dementia in implementing strategies, for example applying what has been discussed during sessions in everyday life. Their involvement could range from very little (e.g. attending brief parts of some sessions) to being present at all times. Phase 2 involves the application of change processes, which the therapist will adapt according to the needs and strengths of the individual. These include identifying and practicing strategies for feeling safe, identifying and challenging unhelpful cognitions, ‘realistic negative automatic thoughts’, calming thoughts (on cue cards) and behavioural experiments. Phase 3 works on ending the therapy and developing a blueprint for the future. This includes reviewing and consolidating learned skills, integration of skills into everyday life and considering the future involvement of carers and others.

Sessions will cover themes and broad ideas, but there will be specific guidelines on how to adapt them to suit the needs of individuals. For example, the degree to which ‘cognitive’ and ‘behavioural’ elements are used will vary, with higher functioning participants being more able to access the ‘cognitive’ and a greater emphasis on behavioural aspects with the more impaired.

For some, sessions may need to be shortened or breaks may be required to maximise attention.

Telephone contact will be offered between sessions to answer questions and encourage ongoing work, should it be required. Sessions will be delivered by clinical or counselling psychologists, with experience of working with people with dementia. They will be given regular clinical supervision by a senior old age clinical psychologist with specialist CBT training.

Treatment as usual (TAU): This is defined as the standard treatment available to people with anxiety and dementia, which is most likely to include medication or no treatment.

#### Outcome measures

All outcome measures will be administered by the main research assistant, blind to group allocation, at week 1 (baseline), week 15 (follow-up 1) and 6 months (follow-up 2). Fifteen weeks for follow-up 1 was determined by the field-testing phase, which suggested that it takes an average of 15 weeks to complete a ten-session intervention because of factors such as randomisation time, illness and holiday. Demographics and general information will be collected including age, gender, ethnic group, use of medication (including anxiolytics and cholinesterase inhibitors), treatment preference and participation in other activities. The Clinical Dementia Rating (CDR) [23] will be used to provide a global rating of dementia severity as part of the general background information.

#### Primary outcome measures

1. **Rating Anxiety in Dementia** (RAID) [24]. This rates signs and symptoms of anxiety using interviews with carers and people with dementia. There are 18 questions in four categories: worry, apprehension, vigilance, motor tension and autonomic hypersensitivity. A score of 11 or above indicates significant clinical anxiety. It has good inter-rater and test-retest reliability, is sensitive to change and correlates with quality of life [25].

2. **Clinical Services Receipt Inventory** (CSRI) [26]. A tool that collects information about the interviewee’s use of health and social care services, accommodation and living situations, income, employment and benefits, and any changes that may be incurred in the receipt of these services (after the therapy).

#### Secondary outcome measures

3. **Mood**: Hospital Anxiety and Depression Scale (HADS) [27]. The HADS is a widely used measure of anxiety and depression validated for all age groups.

4. **Quality of life** (QOL): Quality of Life-Alzheimer’s Disease (QOL-AD) [28]. This is a self-report measure for the person and their carer, with 13 items covering domains including physical health, energy, friends and fun. It has excellent inter-rater reliability and internal consistency, and good content, criterion and construct validity.

5. **Behavioural disturbance**: Neuropsychiatric Inventory (NPI) [29]. This assesses ten areas including delusions, hallucinations, dysphoria and agitation/aggression. Content and concurrent validity, inter-rater and test-retest reliability and internal consistency are all good.

6. **Cognitive function**: Mini-Mental State Examination (MMSE) [30]. This is an internationally recognised, 11-item set of simple tasks presented to the participant including orientation to time and place, attention, recall, language and visual construction. It has a maximum score of 30 points, with 24 or less suggesting cognitive impairment. Reliability and validity are satisfactory.

7. **Depression**: Cornell Scale for Depression in Dementia [31]. This rates depression in five categories including mood-related signs, behavioural disturbance and ideational disturbance, using information from interviews with staff and participants. Good reliability and validity have been demonstrated.

8. **Personcarer relationship**: Quality of Caregiver and Patient Relationship (QCPR) [32]. This is a 14-item scale measuring relationship quality including the level of criticism and level of warmth, rated by both the person and their carer. Good reliability and validity have been demonstrated.

9. **Carer mood**: Hospital Anxiety and Depression Scale (HADS) [27].

#### Qualitative interviews

After follow-up 1, 12 patient-carer dyads who received CBT will be invited to take part in qualitative interviews, led by another research assistant (to ensure blindness to treatment) and lasting approximately 1 hour. Issues addressed will include experiences of the sessions, views on themes and techniques, and benefits/changes identified in everyday life. Interviews will be audio recorded and transcribed. Framework analysis [33] will be used to extract themes and draw conclusions.

#### Treatment implementation strategies

Treatment implementation (TI) strategies are recommended to ensure that treatment has been delivered, received and enacted as intended [34]. We will employ several methods to monitor treatment delivery and receipt: (1) the training and manual; (2) an adherence checklist devised in the manual, developed for the purpose of the study; (3) by audio recording all sessions, of which a random sample will be checked by GC for adherence to the manual. Treatment receipt and enactment will also be explored through qualitative interviews.

#### Statistical analysis

As this is a pilot trial, our main aims in evaluation will be to record response to recruitment, acceptability of randomisation, retention in therapy, attrition, prescribing of anxiolytics, any negative effects, extent/distribution of missing data and basic costs. We will conduct exploratory statistical analyses as our sample size will limit definitive conclusions. Data will be analysed by SPSSv16 using 'intention to treat'. Differences in outcomes between the two arms of the RCT over time will be modelled using generalised estimating equations, taking into account potential clustering due to using several therapists. We will also conduct exploratory analyses of factors predicting treatment outcome such as anxiolytic medication, age, dementia severity, treatment preference and number of sessions received. The confidence intervals around the effect size for the primary outcome, anxiety, will help us to estimate the sample size required for a full-scale trial.

## Discussion

The NHS predominantly offers either nothing (as anxiety frequently goes unrecognised) or medication for anxiety in dementia, even though NHS guidelines suggest that it should only be used if there is severe distress or risk [2]. We will produce a CBT training manual for staff, which we will make available online. This will enable its widespread use by NHS professionals. This trial should prepare the ground for a definitive RCT, with CBT potentially offering an alternative to medication for many people. CBT could lead to significant and generalised benefits, reducing excess disability and social exclusion, by improving cognitive and behavioural functioning and reducing carer burden. Costs to the NHS might be reduced through decreased use of medication, services (such as GP visits) and reduced nursing home placement.

The National Dementia Strategy [35] highlights (1) the need for early treatment and support around the time of diagnosis and (2) keeping people at home for longer. CBT could provide the emotional support and skill development needed at diagnosis, often a time of extreme anxiety, and improve the home situation by offering carers strategies to help the person at difficult times and across situations. Ultimately, it will allow patients the option of a first-line psychological intervention, the norm in general clinical practice.

### Trial status

The trial commenced in November 2010 and will end in April 2013. It is currently half way through the recruitment phase, with 25 of the 50 participants recruited.

## Abbreviations

CBT: Cognitive behavioural therapy; CDR: Clinical Dementia Rating; CSRI: Client Service Receipt Inventory; DoH: Department of Health; HADS: Hospital Anxiety and Depression Scale; IAPT: Improving access to psychological therapies; MMSE: Mini-Mental State Examination; MRC: Medical research council; NELFT: North East London NHS Foundation Trust; NEPFT: North Essex Partnership NHS Foundation Trust; NICE: National Institute of Clinical Excellence; NPI: Neuropsychiatric Inventory; QCPR: Quality of Caregiver and Patient Relationship; QOL: Quality of life; QoL-AD: Quality of life-Alzheimer’s disease; RAID: Rating Anxiety in Dementia; RCT: Randomised controlled trial; SPSS: Statistical Product and Service Solutions; TAU: Treatment as usual; TI: Treatment implementation.

## Competing interests

The authors declare that they have no competing interests.

## Authors’ contributions

AS conceived of the study, led its design and coordination, and drafted the manuscript. MO provided trial expertise. ML prepared materials for the article and contributes to the daily trial management. JH provided admiral nurse input. MK oversaw the methodology. KH provided input from a carer’s perspective. AQ provided psychiatry expertise. GC provided expertise from a CBT perspective. All authors have read and contributed to drafts of the manuscript.
